# Leprosy patients with deformities at post‐elimination stage: The Bangladesh experience

**DOI:** 10.1002/ski2.5

**Published:** 2020-10-14

**Authors:** M. R. Mowla, D. M. Angkur, Z. Hasan, M. N. Sultana, S. Afrin, M. S. Akhter

**Affiliations:** ^1^ Department of Dermatology and Venereology Chittagong Medical College Chittagong Bangladesh

## Abstract

**Background:**

Disability assessment in leprosy patients is a very important factor in the evaluation of the effectiveness of a leprosy elimination program. Little information exists on deformities in leprosy patients in Bangladesh.

**Objectives:**

To describe the pattern and prevalence of deformities in leprosy patients after leprosy has been declared eliminated from Bangladesh in 1998.

**Methods:**

A descriptive retrospective cross‐sectional study was carried out in Chittagong Medical College Hospital using the registered records of patients for the period 2004–2013.

**Results:**

Out of 670 leprosy patients, 213 (31.79%) had deformities. The prevalence of deformity was for grade 1: 92 (43.20%), for grade 2: 121 (56.80%). Among the patients with deformity, males 144 (67.60%) outnumbered females 69 (32.40%). Four age groups were considered. The calculated age‐specific cumulative detection rates showed the highest case detection in >40 years group at 81 (38.02%). The rate of children (<14 years) was less at 7 (3.29%). Of the 213 patients with deformity, the borderline tuberculoid patients were totalled 79 (37.08%), which was higher than other forms of leprosy. Among the 121 patients with limb deformity, 57 (8.50%) had claw hand followed by wrist drop 31 (4.63%), foot drop 30 (4.48%). Three (0.45%) had a nerve abscess, 27 (4.02%) had a trophic ulcer and 7 (1.05%) patients had ocular complications.

**Conclusion:**

The grade 2 deformities among newly detected leprosy patients were still high. Claw hand was the most common deformity in the upper limbs, whereas foot drop and trophic ulcer were the most common deformities in the lower limbs. Although leprosy according to the World Health Organization has been eliminated globally, the disease continues to be a significant cause of peripheral neuropathy, deformity, disability and disfigurement in some developing countries like Bangladesh.

## 1 INTRODUCTION

Leprosy stands out for its morbidity, notwithstanding its low mortality rates, leading to physical disability, deformity, psychological disturbances, economic dependence and social exclusion.^1^ It has been estimated that 2 million people presently live with physical incapacity as a consequence of the disease.^2^ Nerve damage in leprosy is associated with physical disability and deformity and is considered the most severe complication of leprosy.^3^, ^4^, ^5^, ^6^, ^7^, ^8^ The deformities resulting from leprosy cause misery to patient and result in extensive loss of manpower and economic loss to the society.^9^ The term ‘disability’ continues to be used as a synonym for impairments and deformities. Even the World Health Organization (WHO) expert committee on leprosy continues to use ‘disability grading’ while referring to grading of impairments.^10^ Despite emphasis on ‘deformities’ and ‘rehabilitation’, it is surprising to find that relevant information is hardly available. Data on the magnitude of the problem of leprosy‐related impairments are not easily available.^11^ The targets under WHO, Global Leprosy Strategy (2016–2020) are number of children diagnosed with leprosy and deformities are zero, the rate of newly diagnosed cases with deformities are <1 per million and number of countries with legislation allowing discrimination on leprosy are zero.^12^ A progressive increase in grade 2 deformities seems to indicate an increasing delay in the detection of cases, which itself is indicative of operational failure.^13^ In leprosy, the disability assessment is a very important factor in the evaluation of the effectiveness of the National Leprosy Elimination Program (NLEP). There are limited data on deformities of leprosy in Bangladesh. The present study is undertaken to describe the pattern, the prevalence of deformities in leprosy patients after leprosy has been declared eliminated in the year 1998.

## 2 MATERIALS AND METHODS

### 2.1 Study design and setting

The present investigation is a descriptive retrospective cross‐sectional study carried out using the register records of patients attending the leprosy clinic in Chittagong Medical College Hospital (CMCH) during the period 2004–2013. CMCH is the oldest tertiary care teaching hospital of the country. The leprosy clinic of CMCH caters for the patients from the city of Chittagong as well as from neighbouring districts and multi‐drug therapy (MDT) is available at the leprosy clinic of CMCH. The patients' cards were studied and the following clinical data were recorded: age, sex, clinical type of leprosy according to Ridley and Jopling classification,^14^ WHO classification for treatment, leprosy reactions (LRs), smear positivity with bacterial index (BI) and deformity status.

### 2.2 Diagnostic procedures

The diagnosis was made by a specialist based on clinical history, physical examination and laboratory investigations including biopsy for histopathology. After the diagnosis of leprosy, the patient was categorized either as pauci‐bacillary (PB) or multi‐bacillary (MB). The patient was classified as PB if he or she had ≤5 skin patches with or without 1 to 2 thickened major peripheral nerves. Patients with ≥6 patches and/or >2 thickened nerves and those with infiltrations with or without papules or nodules and smear positive were classified MB.

LRs were assessed as type 1 reaction (T1R), type 2 reaction (T2R) and neuritis. Nerve function impairment (NFI) is defined as any reduction in sensory or motor function. The detection of NFI was done clinically. Graded Semmes–Weinstein monofilaments (or a ball‐point pen) were used to detect sensory loss. Voluntary muscle testing was used to assess motor nerve function.

A new case was defined as one ‘who had not been diagnosed earlier and had no history of treatment for leprosy in the past’. Slit skin smear was done to define positive or smear negative cases. Smear positivity was always labelled as MB.

Relapses were diagnosed by supportive information such as clinical course, mode of onset, site of lesions, accompaniment of systemic features, changes (by bacterial test and biopsy) in BI and treatment status if necessary.

### 2.3 Case detection

Mode of detection was categorized as active and passive. Active detection meant that the patients were found by household survey done by health workers of NLEP and passive detection meant that they were either referred by physicians or voluntary reporting by the leprosy patients themselves. The NLFP workers usually carried out the contact tracing (at least 40 household survey) in the area where a new case was detected.

Physical disability was the parameter investigated; all other variables were explanatory. Face, eyes, hands and feet were examined for any visible deformities. The WHO classification of physical disability in leprosy was defined in three categories^15^:

1. No disability (no anaesthesia) and no visible deformity or damage to the eyes, hands or feet (grade 0)
2. Only disability (anaesthesia, but no visible deformity or damage to the eyes, hands or feet; grade 1)
3. Visible deformity or damage to the eyes (lagophthalmos, iridocyclitis, corneal opacities, severe visual impairment), hands (claw hands, ulcers, absorption of the digits, thumb‐web contracture and swollen hand), and feet (plantar ulcers, foot drop, inversion of the foot, clawing of the toes, absorption of the toes, collapsed foot and callosities; grade 2)

The ulcers in leprosy patients were categorized as (1) primary ulcers or specific ulcers and (2) secondary ulcers or non‐specific ulcers. Primary ulcer was defined as caused by *Mycobacterium leprae* and contained *M. leprae*. Secondary ulcer was clarified as not caused by *M. leprae*, but by an injury.

### 2.4 Treatment protocol

All the newly detected patients were put on WHO recommended MDT (dapsone, rifampicin, clofazimine) according to type of disease, 6 months for PB (rifampicin + dapsone), 12 months for MB (rifampicin + dapsone + clofazimine). T1R, including neuritis was treated mainly with a standard 12‐week course of steroids as recommended by WHO with a starting dose of 40 mg/day. T2R was mainly treated with the same dose of 40 mg/day of prednisolone in a tapering regime.

### 2.5 Exclusion criteria

Patients with deformities of some other known cause.

### 2.6 Statistical analysis

Age and gender were analysed separately. The prevalence of deformities was estimated dividing the number of new cases of deformity by the total number of patients in each category. The data were filed and processed using Microsoft Excel software, 2007 version. Data were presented by table, bar diagram, pie chart and line chart, accordingly.

### 2.7 Ethical adherence

The study was approved by our institute's ethics committee.

## 3 RESULTS

**FIGURE 1:**
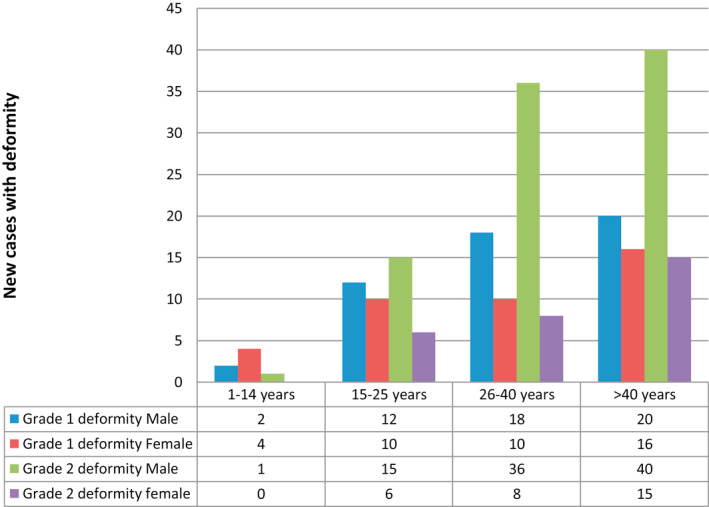
Distribution of leprosy patients with deformity by age and sex

Out of 670 leprosy patients, 213 (31.79%) had a deformity. The case detection was active in 55 (8.21%) patients and was passive in 615 (91.79%). There were 232 (34.63%) PB patients and 438 (65.37%) MB patients. There were only 139 (20.75%) smear positive cases. The prevalence of leprosy reaction was 300 (44.78%) and the prevalence of deformity was 92 (43.20%) for grade 1, 121 (56.80%) for grade 2 (Table 1). Among the patients with deformity due to leprosy, there were 144 (67.60%) males who outnumbered females 69 (32.40%). Four age groups were considered. The calculated age‐specific cumulative detection rates showed the highest case detection in the group >40 years at 81 (38.02%; Figure 1). The rate of children (<14 years) was the least at 7 (3.29%). The youngest patient with a deformity was 8 years old and the oldest patient was 70 years old. Of the 213 patients with deformity, 79 (37.08%) were borderline tuberculoid, which was higher compared to other forms of leprosy (Table 2). Among the 121 patients with limb deformity, 57 (8.50%) had a claw hand followed by wrist drop 31 (4.63%), foot drop 30 (4.48%) and nerve abscess 3 (0.45%). Out of all 670 new leprosy patients, only 27 (4.02%) had a trophic ulcer. A total of seven (1.05%) patients were presented with ocular complication (Table 3).

**TABLE 1:** Patients characteristics (*n* = 670)

Year	New case	Types of patients		Sex		Mode of detection		Deformity		SSS positive	Reactions		
		PB	MB	Male	Female	Active	Passive	Grade 1	Grade 2		RR	ENL	Neuritis
2004	94	46	48	63	31	10	84	11	12	24	21	4	11
2005	65	28	37	50	15	6	59	6	12	8	17	1	8
2006	82	42	40	57	25	9	73	7	12	11	24	2	6
2007	78	28	50	59	19	7	71	9	7	15	18	7	13
2008	81	19	62	54	27	8	73	21	11	11	16	7	10
2009	93	23	70	71	22	7	86	16	21	19	20	9	9
2010	73	25	48	55	18	6	67	7	18	12	20	3	16
2011	26	5	21	22	4	0	26	3	4	11	9	4	4
2012	50	10	40	33	17	1	49	6	16	17	16	6	3
2013	28	6	22	24	4	1	27	6	8	11	5	6	5
Total	670	232	438	488	182	55	615	92	121	139	166	49	85
Percentage		34.63	65.37	72.84	27.16	8.21	91.79	13.73	18.06	20.75	24.78	7.31	12.69

Abbreviations: ENL, erythema nodosum leprosum; MB, multi‐bacillary; PB, pauci‐bacillary; RR, reversal reaction; SSS, slit skin smear.

**TABLE 2:** Prevalence of deformities according to type of leprosy (*n* = 213/670)

Clinical type of leprosy	Grade 1 deformity		Grade 2 deformity		Total (%)
	Male (%)	Female (%)	Male (%)	Female (%)	
TT	18 (8.45)	17 (7.98)	1 (0.47)	1 (0.47)	37 (17.37)
BT	15 (7.04)	12 (5.63)	43 (20.19)	9 (4.22)	79 (37.08)
BB	3 (1.40)	1 (0.47)	5 (2.35)	2 (0.94)	11 (5.16)
BL	2 (0.94)	1 (0.47)	15 (7.04)	3 (1.40)	21 (9.86)
LL	3 (1.40)	1 (0.47)	28 (13.14)	14 (6.57)	46 (21.60)
PN	11 (5.16)	8 (3.75)	0	0	19 (8.92)
Total	52 (24.41)	40 (18.78)	92 (43.19)	29 (13.61)	213 (100.0)

Abbreviations: BB, borderline; BL, borderline lepromatous; BT, borderline tuberculoid; LL, lepromatous; PN, pure neural; TT, tuberculoid.

**TABLE 3:** Distribution of deformities (*n* = 670)

Deformity	Presentation	Frequency	Percentage
Limb deformity	Claw hand	57	8.50
	Wrist drop	31	4.63
	Foot drop	30	4.48
	Nerve abscess	3	0.45
Ocular complications	Lagophthalmos	1	0.15
	Keratitis	2	0.30
	Pain or discomfort	1	0.15
	Photophobia	1	0.15
	Dimness of vision	2	0.30
Ulcers	Primary ulcer	21	3.13
	Secondary ulcer	6	0.89

## 4 DISCUSSION

The aim of this study was to find the pattern, prevalence of deformities in leprosy patients in the post‐elimination era. For understanding the current magnitude of the problem, the study will be helpful in redesigning NLEP and to reduce the deformity rate among the newly detected leprosy cases. Disability assessment is important not only to evaluate the effectiveness of the control program but also for the patient, whose most important worry are the stigmatizing deformities.

There were 144 (67.60%) male and 69 (32.40%) female patients with a sex ratio of 2.08:1. This is similar to Indian studies where Mangala HC et al. reported males 63.71%, females 36.28% and Sankar A et al. found male 69%, females 31%.^16^, ^17^ This is explained by the assumption that women in general are poorly represented in hospital statistics due to socio‐economic and cultural difficulties. If this is true, the lack of recruitment of women is a cause for concern. The higher incidence of deformities in males could be attributed to the higher prevalence of leprosy among males and another explanation could be that males are more likely to be engaged in heavy physical activities where the risk of deformity seems to be increasing.

**FIGURE 2:**
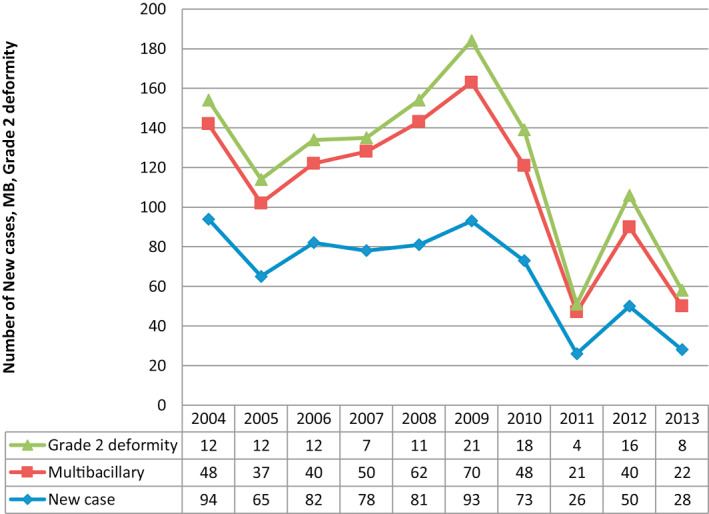
Year‐wise trend of leprosy with deformity

Out of all 670 leprosy patients, 121 (18.06%) were presented with grade 2 deformities and 57 (8.50%) had claw hand followed by wrist drop 31 (4.63%), foot drop 30 (4.48%) and nerve abscess 3 (0.45%). Indian studies also reported the claw hand to be the highest at 32.74%, 38% and 60%.^17^, ^18^, ^19^ There was a high presence of deformities in the year 2009 compared with other years, which could be explained by intensified contact tracing that year. The year‐wise trend of grade 2 deformities is in upward trend and is similar to Bangladesh national scenario (Table 4 and Figure 2). The high prevalence of deformities may reflect a failure of the leprosy services to detect new patients' timely, inadequate patient management due to resource constraints and patient's non‐compliance to MDT. There may also be a delay in seeking treatment and even after identifying symptoms due to lack of health education and hygiene, and sometimes due to the patients' reliance on non‐medical, herbal and traditional healers. Most of the patients belonged to the lower socio‐economic class. It is highly possible that new cases were detected late owing to inadequate community awareness of consequences of the disease and lack of possibilities to attend the leprosy services. A good surveillance is required to detect leprosy and to ensure good patients' compliance with treatment to prevent deformity and disability. Information campaign about leprosy in high risk areas are crucial, so that patients and their families who are historically ostracized from their communities are encouraged to come forward to receive treatment.^20^

**TABLE 4:** Comparative year‐wise prevalence rate at CMCH and national level

	CMCH			National		
Year	New case	MB (%)	Grade 2 deformity (%)	New case	MB (%)	Grade 2 deformity (%)
2004	94	48 (51.06)	12 (12.76)	8242	2885 (35.0)	561 (6.81)
2005	65	37 (56.92)	12 (18.46)	7883	3018 (38.28)	650 (8.25)
2006	82	40 (48.78)	12 (14.63)	6281	2389 (38.04)	525 (8.36)
2007	78	50 (64.10)	7 (8.97)	5357	2347 (43.81)	556 (10.38)
2008	81	62 (76.54)	11 (13.58)	5249	2350 (44.8)	616 (11.74)
2009	93	70 (75.27)	21 (22.58)	5238	2386 (57.3)	542 (10.30)
2010	73	48 (65.75)	18 (24.66)	4183	1720 (41.19)	468 (11.19)
2011	26	21 (80.77)	4 (15.38)	3968	1804 (45.46)	480 (12.10)
2012	50	40 (80.0)	16 (32.0)	3386	1926 (56.88)	388 (11.46)
2013	28	22 (78.57)	8 (28.57)	3141	1380 (43.94)	341 (10.86)
Total	670	438 (65.37)	121 (18.06)	52918	22205 (41.96)	5127 (9.69)

Abbreviations: CMCH, Chittagong Medical College Hospital; MB, multi‐bacillary.

Among the eye complications keratitis (0.30%), dimness of vision (0.30%), lagophthalmos (0.15%), photophobia (0.15%) and eye pain (0.15%) were recorded. Other studies from India reported two (1.76%), three (6%) patients respectively with lagophthalmos.^17^, ^18^ A study from the United Kingdom found highest diminished lid closure (19%) and mild corneal opacity (13.5%).^21^ Ocular morbidity and blindness can be a devastating complication for leprosy patients who may rely on their eyes to protect their anaesthetic limbs. It seems to be a more serious impairment than those involving hands or feet. Ocular involvement is a danger sign, even in early stages before it causes significant dimness of vision, particularly when both eyes are affected. Ocular morbidity distresses and incapacitates the affected person seriously and sometimes diagnosis can be delayed because of corneal insensitivity masking some of the symptoms. Any delay in diagnosis would contribute to more severe ocular complications and blindness. There is a need for good baseline ophthalmological examination for all leprosy patients. The patient should be advised to promptly seek ophthalmological care for any new eye symptoms to prevent avoidable blindness, due to the life‐long risk of sight‐threatening ocular complications.

A total of 21 (3.13%) patients had a primary ulcer and six (0.89%) had secondary ulcer. Ulcers may also be considered as serious or potentially serious impairments, in view of their propensity to further cripple the person. As with the other impairments, reliable information is not easily available about the prevalence and incidence of plantar and palmar ulcers. A primary impairment is a direct consequence of the causative disorder (e.g., madarosis, collapse of nasal bridge) and secondary impairment is not caused directly from the original disorder (e.g., trophic ulcer).^22^ The impairments seen in leprosy‐affected persons range from mild such as a small area of anaesthesia on the hand, to a very severe degree such as shortening of fingers and thumbs in both hands, claw hands, bilateral wrist drop, ulceration and fixed deformities of both feet rendering them useless for walking, and loss of vision in both eyes. The milder ones are more common. However, grade 2 impairments do not arise de novo, and it is usually the patients with grade 1, with only anaesthesia, who develop grade 2 impairments. The earliest detection of sensory loss might reduce these secondary deformities. Timely diagnosis of grade 1 deformity is required for disability limitation and mitigation. Therefore, deformity is a preventable complication in the majority of patients. Many patients can develop deformity after being diagnosed or after starting treatment, post‐reactions or even after release from treatment. In the post‐elimination period, there is a lack of training to assess deformity and progressively monitor patients for further deformity post‐treatment. These are issues that need to be addressed after being released from treatment (RFT) to ensure the quality of living a meaningful life. One must realize that leprosy infected person does not die, the consequences of the damage will stay with the patient all his life. It will contribute to his or her disability, will diminish the quality of life and will increase the fear in his or her surroundings. We need to diminish this fear and turn it into cooperation because only then we will be able to achieve zero disability in girls and boys.^23^

Risk factors for developing grade 2 deformity were analysed as follows: MB cases (77.69%), male (76.03%), age >40 years (45.45%), >6 skin lesions (79.33%), >2 nerves trunk involvement (88.43%), high bacillary index and leprosy reactions (Table 5). This is in agreement with other studies conducted in different parts of the world.^18^ ^,^ ^24^, ^25^, ^26^, ^27^, ^28^, ^29^

**TABLE 5:** Risk factors for developing grade 2 deformity (*n* = 121/670)

	Type of leprosy		Age				Sex		Bacterial index		Extent of clinical presentations	
Leprosy patients with deformity	PB	MB	1–14 years	15–25 years	26–40 years	>40 years	Male	Female	<3–1	≥3	≥6 skin lesion	≥2 nerves involvement
Total	27	94	1	21	44	55	92	29	15	34	96	107
Percentage	22.31	77.69	0.83	17.36	36.36	45.45	76.03	23.97	12.40	28.09	79.33	88.43

Abbreviations: MB, multi‐bacillary; PB, pauci‐bacillary.

The major limitation of the study is the fact that nearly all the prevalence and case finding figures are derived from hospital attendance. The single prevalence rate does not reflect the real situation. Therefore, a multi‐centre study across the country in concurrence with large population based survey is recommended. Although it is hospital‐based and large number of patients over a long period of study involved, it provides a rough indicator which can serve as a baseline upon which future studies can be built for effective planning of patient oriented leprosy services and prudent allocation of scarce resources.

## 5 CONCLUSION

The grade 2 deformities among newly detected leprosy patients still occur high. Claw hand was the most common deformity in the upper limb, whereas foot drop and trophic ulcer were the most common deformities in the lower limb. Although leprosy has been eliminated globally on study, the disease continues to be significant cause of peripheral neuropathy, deformity, disability and disfigurement in some developing countries like Bangladesh. Community‐based surveillance could help to improve early detection, treatment, case holding and prevention of deformities and stigma. Patient should be educated to undergo regular follow‐up examination and self‐care along with their family members to prevent deformity. Rehabilitation services to be maintained also after RFT to improve the quality of life index and to raise the self‐esteem of the affected persons as well.

## CONFLICT OF INTERESTS

No conflict of interests have been declared.
